# Neurophysiological and behavioral correlates of alertness impairment and compensatory processes in ADHD evidenced by the Attention Network Test

**DOI:** 10.1371/journal.pone.0219472

**Published:** 2019-07-25

**Authors:** Dimitri M. Abramov, Carla Quero Cunha, Paulo Ricardo Galhanone, Renata Joviano Alvim, Andrei Mayer de Oliveira, Vladimir V. Lazarev

**Affiliations:** 1 Laboratory of Neurobiology and Clinical Neurophysiology, National Institute of Women, Children and Adolescents Health Fernandes Figueira, Oswaldo Cruz Foundation, Rio de Janeiro, Brazil; 2 Department of Physiological Sciences, Center of Biological Sciences, Federal University of Santa Catarina, Florianopolis, Brazil; Sorbonne Universite, FRANCE

## Abstract

In Attention Deficit Hyperactivity disorder (ADHD), fMRI studies show asymmetric alterations: widespread hypoactivation in anterior cortical areas and hyperactivation in some posterior regions, and the latter is considered to be related to compensatory processes. In Posner’s attentional networks, an important role is attributed to functional interhemispheric asymmetries. The psychophysiological Attention Network Test (ANT), which measures the efficiency of the alerting, orienting, and executive networks, seems particularly informative for ADHD. Potentials related to ANT stimuli (ANT-RPs) have revealed reduced cognitive potential P3 in ADHD. However, there are no studies associated with asymmetry of ANT-RPs. In the present study, conducted with 20 typically developing boys and 19 boys with ADHD, aged 11–13 years, the efficiency of the three Posner’s networks regarding performance and amplitude asymmetries in ANT-RPs was evaluated according to the arithmetic difference of these parameters between different cue and target presentation conditions. The results were correlated to Diagnostic and Statistical Manual of Mental Disorders (DSM-IV-TR) scores. Regarding accuracy and intraindividual variation in reaction time, ADHD subjects showed lower efficiency of executive and alerting network, and this effect was correlated with DSM. Regarding alerting network, ANT-RPs in ADHD did not have the right-side amplitude prevalence in the temporal regions, which was observed in controls. In all ANT conditions, significantly higher asymmetries were observed in ADHD than in controls in the occipital regions 40–200 ms after target onset. Their amplitude in ADHD subjects was inversely proportional to DSM scores of inattentiveness and directly proportional to accuracy and efficiency of the executive network. The results suggest impaired alerting and executive networks in ADHD and compensatory occipital mechanisms.

## Introduction

Attention Deficit Hyperactivity disorder (ADHD) is one of the most prevalent cognitive disorders, occurring in approximately 5% of children [1], and is characterized, according to the American Psychiatric Association [2, 3], by two symptom dimensions, related to inattention and hyperactivity with impulsivity. The core of ADHD phenomenology has been related to impaired executive functions due to fronto-striatal-cerebellum changes [4–7]. A review of fMRI studies has indicated widespread hypoactivation in ADHD, bilateral in fronto-parietal areas, especially in the frontal lobes, correlated to executive attentional deficits. Simultaneously, there has been hyperactivation in some regions, such as in the right parieto-occipital areas, considered to be compensatory processes [8]. It has been suggested that some mechanisms in the visual and motor systems compensate verbal/working memory impairments and greater deficit in inhibition/conflict resolution, respectively [9].

In the psychophysiological model for estimating attention known as Posner’s attentional networks [10,11], the executive and alertness functions are segregated as parts of a multidimensional attentional system divided in at least three neural systems, which: (a) maintain vigilance after a phasic change in alertness (“alerting” network) evoked by a warning signal; (b) orient to sensory events by modality or location (“orienting” network); and (c) drive and monitor perception and action by top-down control (“executive” network). These networks can be tested simultaneously using the Attention Network Test (ANT), which is an adaptation of previously developed experimental stimulus-reaction paradigms to test alertness and orientation of the covert attention by cueing, as well as to estimate interference of target information in conflict resolution based on flanker congruency [12]). Using ANT, the efficiency of each network can be measured by the arithmetic difference in performance scores of motor reaction time (RT) related to different ANT conditions (see methods) and there was statistical independence between those differences. A user-friendly ANT version [13] has shown impaired efficiencies of the executive [14–17] and the alerting networks [15, 17], with higher RT and/or IVRT in ADHD subjects.

The functional independence of Posner’s attentional networks was emphasized by their relation to different neurotransmitter systems [10, 11, 18]. This relation may well be seen in the pharmacological treatment of ADHD subjects. Traditional medications for ADHD, such as methylphenidate, predominantly target the dopaminergic system [19]. However, impaired attentional alerting function in ADHD [15,17,20] has been shown to be modulated by noradrenaline [19], and atomoxetine, a selective inhibitor of noradrenaline reuptake, shows an efficacy comparable to methylphenidate [21–24], which is not known to be specific for the above-mentioned neurotransmitter systems.

In Posner’s attention model, an important role is attributed to the functional asymmetries of the brain, and the alerting network is supposed to be related to the right hemisphere [10]. However, such lateralization is still under discussion [11, 25–28]. Structural and functional asymmetries related to ADHD phenomenology have been found in the lateral and medial prefrontal cortex and in the striatum [5, 6, 29, 30], including the above-mentioned interhemispheric differences in compensatory processes [8].

There are no studies in literature on the functional or morphological interhemispheric asymmetry considering Posner's attentional networks measured by ANT. The methodological problem was resolved by combining ANT and the recording of brain event-related potentials (ERPs) evoked by ANT stimuli, i.e., ANT-related potentials (ANT-RPs) [31–33]. The validity of this method for the neurophysiological study of ADHD was emphasized by identifying reduced amplitudes of cue and target P3 in children [31, 32] and also in adults with ADHD [33]. However, the interhemispheric asymmetries were not considered. The neurophysiological estimation of ANT-RPs seems particularly valuable and biologically justified for ADHD, since the neurotransmitter independence of Posner's networks [11] is well manifested in the treatment of ADHD symptomatology [17, 19, 23].

In the present ADHD study, we used ANT to search for: (1) evidence of impaired efficiency of the alerting network of performance characteristics and their correlation with Diagnostic and Statistical Manual of Mental Disorders (DSM) scores; (2) putative alterations in functional asymmetry; and (3) assumed compensatory processes related to Posner’s networks (particularly the alerting network) as reflected in performance parameters and interhemispheric differences in ANT-RP amplitude and their correlation with DSM scores.

## Methods

### Design and volunteer selection

This study was approved by the Ethics Committee of the Fernandes Figueira National Institute (CAAE 08340212.5.0000.5269). All participants gave their oral assent in the presence of their caregivers, who provided written informed consent after a complete description of the study.

Sixty boys aged 10–13 years were recruited, classified, and included in the sample according to the DSM-IV-TR (DSM Text Revision) [2]: 35 with ADHD and 25 typically developing subjects The exclusion criteria were: (1) estimated intelligence quotient (I.Q.) lower than 80 according to the abbreviated version of the WISC-III [34, 35] (five subjects), (2) having taken any psychotropic drugs for the last 30 days (five subjects), (3) chronic diseases, any suspicion of major psychiatric disorders (psychosis, major depressive and bipolar disorders, obsessive-compulsive and tic disorders, phobic and post-traumatic stress conditions) as well as anorexia, bulimia, encopresis or enuresis, as screened by K-SADS-PL [36] (six subjects); (4) less than 6 hours of sleep on the previous night (two subjects); and (5) having manifested improper RT/accuracy tradeoff (see below) (six subjects). Two subjects refused to complete the ANT. As a result, 19 boys with ADHD (11.53 ± 1.07 years old, 5 left-handed) and 20 typically developing boys (11.3 ± 0.86 years old, 5 left-handed) in the control group were selected for this study. All boys with ADHD had not been medicated and were referred to us by clinical specialists from outside our institute. To establish the diagnosis of ADHD, the subjects were evaluated jointly by a psychiatrist and a neuropediatrist in an interview based on the DSM criteria and associated with the application of these criteria to the children’s parents as described below.

Each caregiver was asked to consider DSM-IV-TR criteria for ADHD, one by one, and was instructed to indicate carefully which criteria were notable characteristics of their dependents. Criteria about which caregivers had doubts, showed hesitation, or to which they attributed little emphasis in describing the behavior of the child were not counted. Throughout the clinical interview, we inserted variables which were irrelevant for the diagnosis but could potentially create observation biases (confounding variables), namely: years of study, number of hours on the computer or playing video games per week (1 = less than 2h/week(w); 2 = 2-4h/w; 3 = 5-8h/w; 4 = 8-15h/w; 5 = more than 15 h/w; which could help develop non-specific ability for RT tests), monthly family income, and hours of sleep the previous night. Age was also considered a potential confounding variable. The confounders were decided *a priori*.

### Experimental procedures

ANT is a combination of tests to assess performance in terms of RT, IVRT, and response accuracy (AC), regarding (1) modulation of alertness state under the effect of a Neutral cue (NtCue) compared to absence of cue (NoCue), when the context is under the effect of NtCue just as a warning stimulus that indicates the imminence of target onset; (2) dynamics of attention orientation by assessing the influence of a Spatial cue (SpCue) on NtCue; and finally, (3) the ability to detect and resolve conflicts by assessing the interference of incongruent target distractors on performance parameters compared to congruent target distractors.

**Fig 1:**
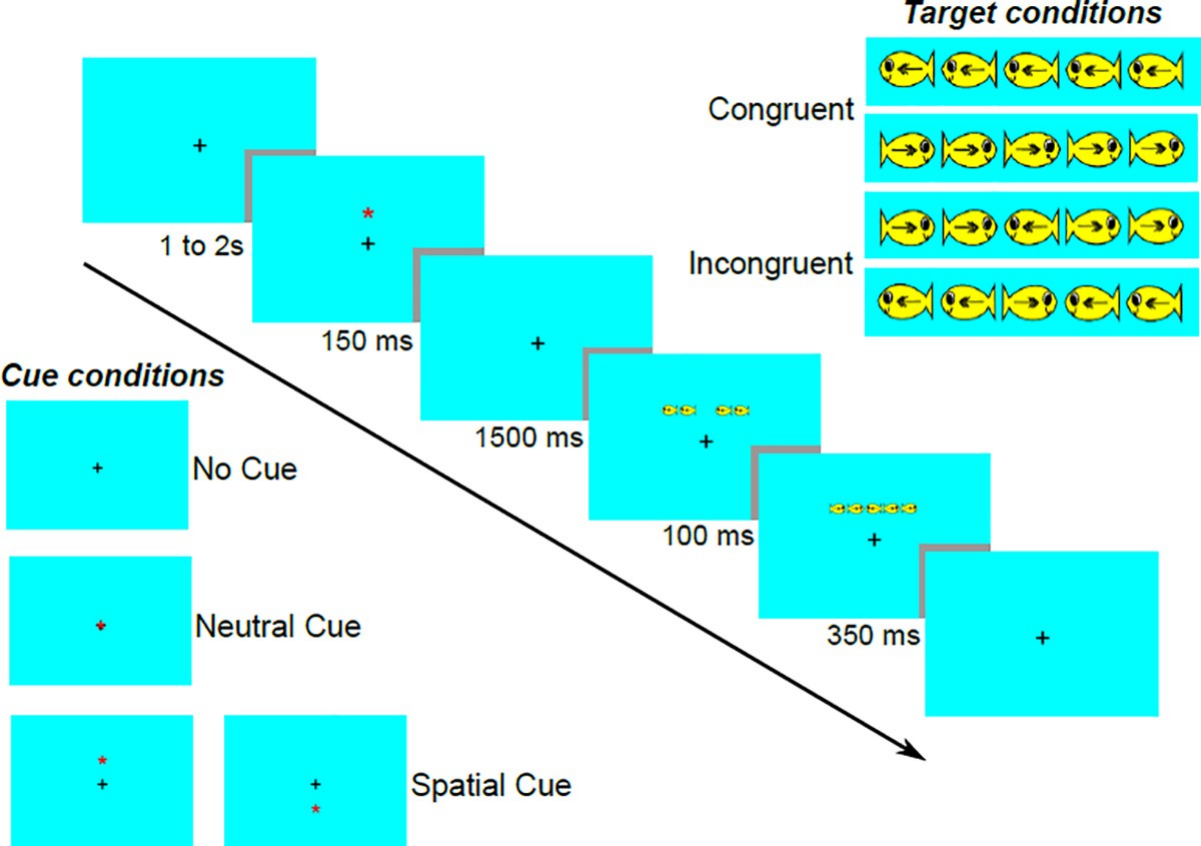
Sequence and duration of signals in an Attention Network Test trial. Arrow: time axis with signal duration (ms); black cross: fixation point; red star: cue signal; central yellow fish: target signal; lateral pairs of yellow fishes: flanks.

We used an ANT version adapted to children [31, 32, 37], with three equiprobable cueing conditions (NoCue, NtCue, and SpCue) operating on a cyan background with a central black cross as a fixation point. The cue (red asterisk) would appear for 150 ms in the center (NtCue) or in the upper or lower hemifields of the screen, above the position where the target would appear afterwards (SpCue), with a stimulus-onset asynchrony of 1650 ms. The target was the picture of a yellow fish facing right or left, which appeared for 350 ms, flanked by distractors: pairs of identical fish, all with the same orientation as the target (congruent condition) or a different orientation from the target (incongruent condition), which appeared 100 ms before the target. The images of signals and their location on the screen, sequence, and duration are shown in Fig 1. The subjects indicated the direction of the target using the left and right keys on the keyboard with their middle and index fingers of the dominant hand as soon as they saw it, keeping a nasion-screen distance of 45 cm.

A total of 24 trials were shown randomly (eight for each cueing condition associated to twelve trials for each target condition) in each one of the nine blocks that comprised the ANT test. The first block was designed for training the subject, and this performance was not counted. Thus, each subject performed a total of 64 trials for each cueing condition and 96 trials for each target condition. The trials were separated from each other by a random gap between 1 and 2 seconds. Each trial was completed once the subject responded to the target signal or after 2 seconds if the subject did not respond. By the end of each block, the participant was given a performance feedback (mean reaction time and number of hits) with motivational sentences indicating improved performance (if any). The subjects started the new block pressing the space bar on the computer keyboard. Accuracy (rate of correct responses), reaction time (RT, ms) and its standard deviation, and termed intraindividual variation (IV) were recorded.

### EEG acquisition

During the ANT performance, the subjects' EEG were acquired using a Nihon Kohden NK1200 EEG System at 20 scalp points according to the International 10/20 System, with linked biauricular reference (A1+A2) at a sampling rate of 1000 Hz and resolution of 16 bits, with low-pass, high-pass, and notch filters at 100, 0.5, and 60 Hz, respectively, under impedances below 10 kΩ. Low frequency movement artifacts were suppressed by high-pass filtering. Muscular artifacts of high amplitude and frequency were removed manually from ongoing EEG signal during post-recording visual inspection. We also used a statistical filter developed in our laboratory to mitigate blinks [38].

### Behavioral and EEG data processing

We determined ANT behavioral variables such as (1) AC—correct responses/total responses, (2) RT, and (3) IVRT. These variables were determined for each ANT condition and for all conditions indistinctively (AllCd). Each variable was analyzed in the set of AllCd to obtain an “overall mean value” of behavioral measures without considering specific attentional dimensions. Speed-accuracy tradeoff was estimated by multiplying AC by RT for all conditions. Subjects with values lower than the mean group value minus two standard deviations were removed from the sample.

The efficiency of the alerting, orienting, and executive (conflict resolution) attentional networks related to behavior and neural functioning were calculated as the difference between behavioral scores (RT, IVRT, and AC) or ANT-RP parameters of the corresponding conditions: [NoCue minus NtCue], [NtCue minus SpCue], and [‘incongruent target distractors’ minus ‘congruent target distractors’] [31, 32].

EEG signals were resampled at 600 Hz using a polyphase anti-aliasing filter to reduce computational load. We calculated ANT-RPs for each cueing and target conditions, as well as their grand average for AllCd. The signals analyzed lay within the range encompassing 1550ms before to 1000 ms after target onset, thus including all ANT-RPs. In addition, we calculated the difference in ANT-RPs between hemispheres, subtracting the signals from homologous channels (right minus left) related to ANT-cue and target conditions. We also subtracted asymmetries (interhemispheric differences) related to different ANT conditions to determine their relation to the efficiencies of the alerting, orienting, or executive networks.

**Fig 2:**
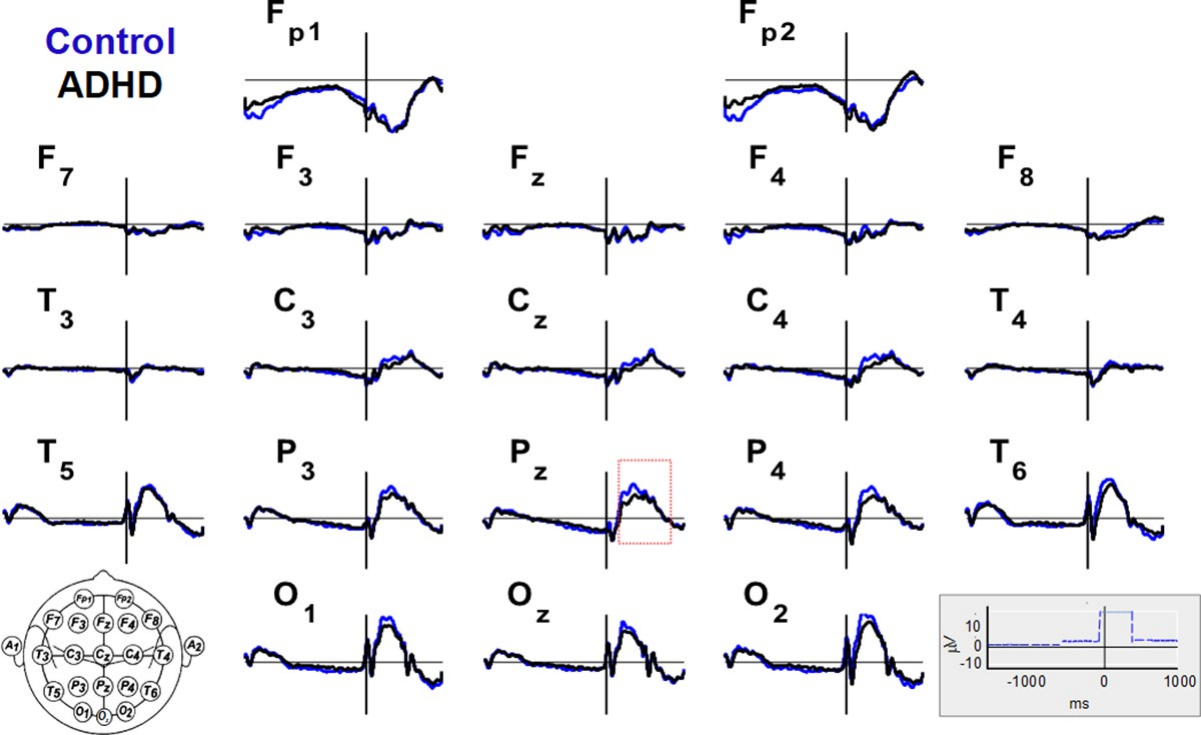
Grand ANT-related potentials averaged across subjects, of all cue and target conditions. Twenty EEG channels in the control (blue) and ADHD (black) groups (scales in the lower right corner, where the blue dashed line is the trigger signal). The red dotted rectangle defines the interval in which the P3 wave is identifiable.

Among all ANT-RPs, we extracted the peak amplitude of late ANT-RPs from Pz channels, which corresponded to P3 waves related to each cue, target, and AllCd condition (dotted box in Fig 2). In the signal resulting from the ‘subtraction of hemispheres’ corresponding to asymmetries in these ANT conditions and their respective network effects, we selected some intervals of interest among which we subtracted NtCue from NoCue to determine the efficiency of the alerting network over asymmetries, and then, we calculated mean amplitudes.

### Statistical analysis

Differences in maximum amplitudes of ANT-RPs between groups, or in mean signal amplitudes within intervals of interest; DSM scores for ADHD diagnosis (inattention, hyperactivity+impulsivity, and total) as well as ACs, RTs, IVRTs were inferred using tests for independent samples. According to the analysis of normality (Shapiro-Wilk test) and homogeneity of variance (Levene Test), we decided to use either parametric (Student t-test) or non-parametric tests (Mann-Whitney U Test), adopting α = 0.05. To evaluate whether the ADHD and TD groups are different as a whole, regarding the parietal P3 wave and the asymmetries in O2-O1 and C4-C3, we corrected p-values using the FDR method of Benjamini-Hochberg.

To study correlations between variables, we sometimes grouped all 39 subjects without considering an *a priori* diagnosis, and we sometimes considered the groups separately. In the first case, we used Pearson's Test, calculating the coefficient of correlation ('r'). For correlations considering the groups distinctively (ADHD, n = 19, and controls, n = 20), we used Spearman rank test to obtain their coefficient of correlation (ρ) assuming the non-normality of samples, *a priori*, because of the small size of the groups. We defined the effect size of correlations according to the following intervals between coefficient values: from 0.0 to 0.09—no correlation; from 0.11 to 0.30—weak correlation; from 0.31 to 0.50—moderate; > 0.50 strong correlation [39]. When interpreting the results, we prioritized coefficient values over their p-values.

**Fig 3:**
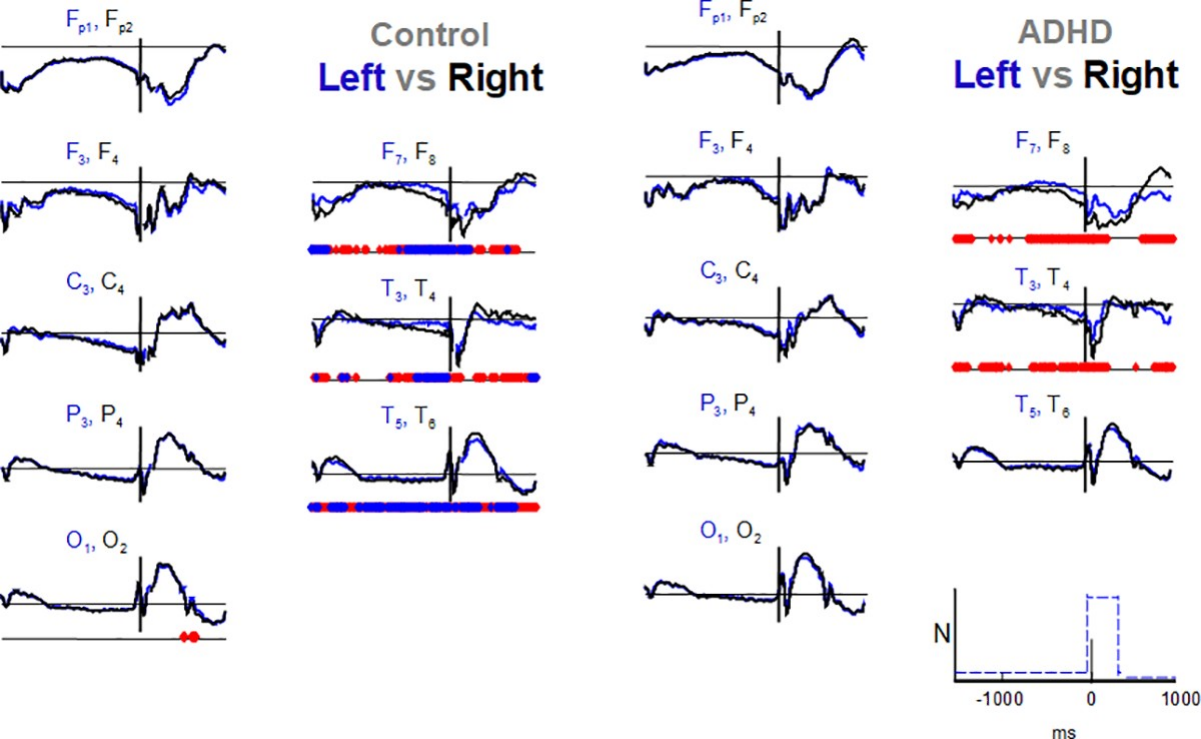
Comparisons of the ANT-related potentials between hemispheres, for all conditions. Blue and black for left and right hemispheres, respectively, by groups, and amplitudes are normalized (N at scales in the lower right corner, positive values above abscissae). Significance level of point-to-point comparisons with FDR-corrected scanning is marked on the parallel abscissae (time axis) below the graphs of each wave pair (blue rhombs, p ≤ 0.01; red rhombs, p ≤ 0.05). See Fig 2 for channel labeling.

**Fig 4:**
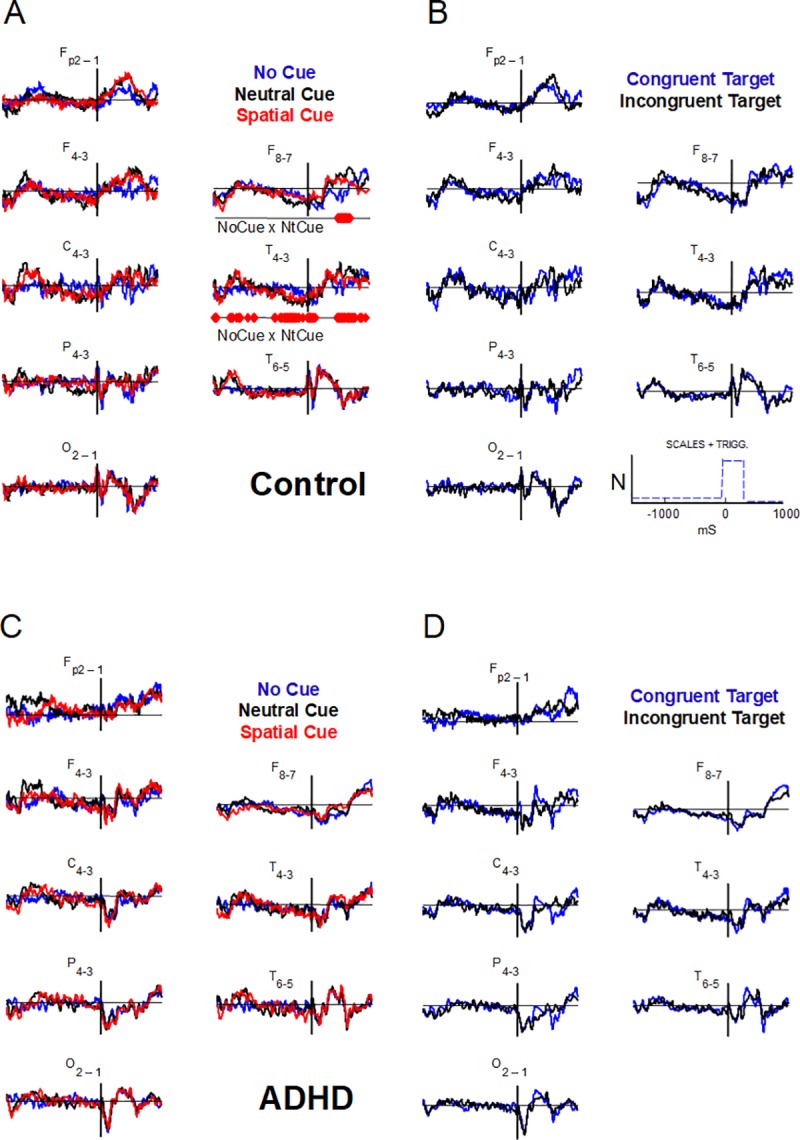
Comparisons of interhemispheric differences in ANT-related potentials between ANT conditions. Each wave is the respective interhemispheric difference (right minus left) of ANT-related potentials for No cue (NoCue, blue), Neutral cue (NtCue, black), and Spatial cue (red) conditions, in A and C, and for Congruent (blue) and Incongruent (black) target conditions, in B and D. Point-to-point statistical comparisons (FDR corrected) between consecutive conditions (NoCue x NtCue, NtCue x SpCue, congruent x incongruent, related to alerting, orienting, and executive networks, respectively). Control groups in A and B, ADHD groups in C and D. Amplitudes are normalized (positive values above abscissae). ‘NoCue x NtCue’–comparison that showed significant differences. See Fig 2 for channel labeling.

**Fig 5:**
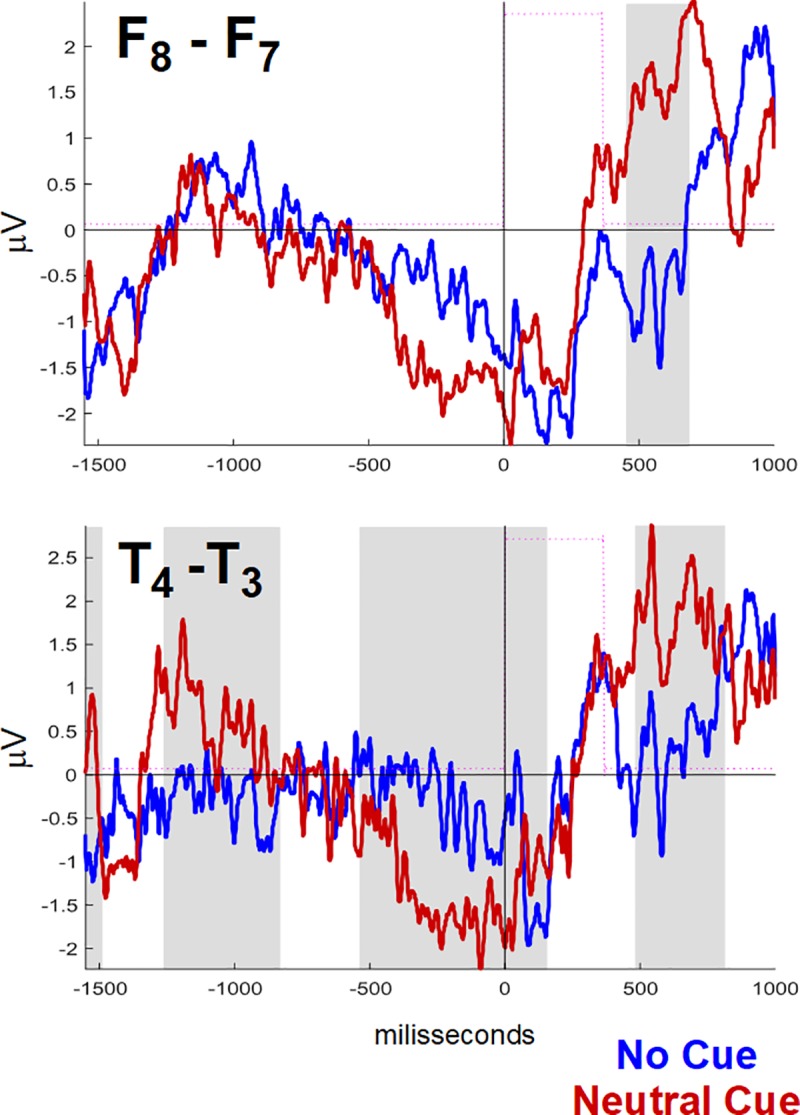
Comparisons of interhemispheric differences of the ANT-related potentials for NoCue and NtCue conditions in control group. Detail from Fig 4 highlighting the waves in anterior- (F8-F7) and mid-temporal (T4-T3) regions; grey background marks time windows where significant differences were found.

To estimate significant statistical differences between hemispheres (Fig 3 and S1 Fig), as well as effects of attentional networks on asymmetries (Figs 4 and 5), we calculated these differences over time (point-to-point) set between a pair of signals (1550 ms before to 1000 ms after target onset). We tested the differences using the non-parametric Wilcoxon Rank Test for paired samples (within each group) in this scanning.

To minimize type-I errors, we adjusted p-values using the positive False Discovery Rate (FDR) correction method [40]. We graphically represented the results using red rhombs (corrected p ≤ 0.05) and blue rhombs (corrected p ≤ 0.01) for each position in time, on parallel abscissae (time axis) below the graphs of each wave pair (Figs 3, 4 and 5).

TIn the S1 Dataset, there is a MatLab/Octave structure with all waves obtained in this study. In the S2 Dataset, there are the tables with DSM, WISC and ANT scores, the data for confounding variables by subject, as well as the peak and mean amplitudes for P3 waves and asymmetries, respectively.

## Results

### Behavioral and psychological data

Among confounding and descriptor variables (Table 1), only estimated IQ was different between groups, with prevalence of the control group (109.4 ± 13.5) over ADHD (97.4 ± 12.5, p = 0.007). In the group with all 39 subjects, this variable had moderately negative correlation with the total DSM score (r = -0.35, p(r) = 0.026), as well as with the inattention score (r = -0.45, p(r) = 0.003), and it did not correlate with hyperactivity+impulsivity scores. DSM scores did not have any significant correlation with any other confounding variables (S1 Table).

**Table 1:** Comparisons between groups of DSM scores, confounder variables, and eficiencies of the attention networks.

Variable		Control			ADHD			p (Levene)^c^	Statistics^d^	p-value
		Mean	SD^a^	p (ShW)^b^	Mean	SD	p (ShW)			
DSM scores										
**Inattention**		**2.40**	**1.67**	**0.055**	**7.21**	**1.27**	**0.006**	**0.053**	**Z = 5.25**	**<0.001**
Hyperactivity + Impulsivity		2.60	1.64	0.057	4.37	2.91	0.479	0.006	Z = 1.95	0.052
**Total**		**5.00**	**2.75**	**0.313**	**11.63**	**3.00**	**0.122**	**0.683**	**T = -7.19**	**<0.001**
Confounder										
Age		11.30	0.86	0.002	11.53	1.07	0.027	0.162	Z = 0.72	0.471
Hours of sleep		7.25	2.12	0.313	7.68	1.73	0.002	0.244	Z = 0.69	0.492
Videogame Sc.^e^		3.40	1.23	0.000	2.89	1.66	0.002	0.103	Z = -0.92	0.359
Computer Sc.^f^		3.30	1.26	0.000	2.95	1.68	0.003	0.113	Z = -0.56	0.574
Years of study		6.05	1.15	0.003	6.11	1.33	0.043	0.433	Z = 0.54	0.590
Familiar incomes		6355.00	5643.58	0.005	3978.95	4436.91	0.000	0.081	Z = -1.49	0.136
**Estimated I.Q.**		**109.35**	**13.54**	**0.548**	**97.37**	**12.53**	**0.447**	**0.520**	**T = 2.86**	**0.007**
ANT behavioral scores										
**Accuracy**	**All conditions**^**g**^	**0.93**	**0.10**	**0.000**	**0.90**	**0.08**	**0.032**	**0.726**	Z = **-2.06**	**0.040**
	Alerting ^h^	-0.01	0.05	0.182	0.01	0.04	0.560	0.972	T = -1.51	0.140
	Orienting	0.02	0.07	0.007	0.00	0.04	0.510	0.368	Z = -0.63	0.532
	**Executive**	**-0.06**	**0.06**	**0.000**	**-0.11**	**0.08**	**0.015**	**0.018**	Z = **-2.14**	**0.032**
Reaction Time	All conditions	0.56	0.12	0.048	0.61	0.14	0.327	0.283	Z = 1.14	0.255
	Alerting	0.02	0.02	0.510	0.02	0.04	0.670	0.030	Z = 0.18	0.855
	Orienting	0.02	0.04	0.584	0.02	0.04	0.266	0.788	T = 0.07	0.943
	Executive	0.11	0.05	0.214	0.14	0.12	0.018	0.069	Z = 0.32	0.747
**IVRT** ^**i**^	**All conditions**	**0.16**	**0.06**	**0.157**	**0.23**	**0.11**	**0.270**	**0.044**	Z = **2.04**	**0.042**
	**Alerting**	**0.18**	**0.06**	**0.576**	**0.30**	**0.15**	**0.274**	**0.014**	Z = **2.68**	**0.007**
	Orienting	0.20	0.09	0.001	0.30	0.17	0.090	0.030	Z = 1.73	0.084
	**Executive**	**0.19**	**0.08**	**0.290**	**0.29**	**0.15**	**196**	**0.022**	Z = **2.37**	**0.018**

^a^ SD–standard deviation

^b^ p-value related to Shapiro-Wilk Test for normality, applied in each group, by variable

^c^ p-value related to Levene Test for homogeneity of variances, applied in each group, by variable

^d^ Test statistics for independent samples: T-statistics from Student t-test, Z-statistics from Mann-Whitney U-test.

^e^ Videogame score: 1—less than 2 hours/week (h/w); 2–2–4 h/w; 3–5–8 h/w; 4–8–15 h/w; 5—more than 15 h/w

^f^ Computer score: see comment 4

^g^ Scores for all ANT conditions

^h^ Scores for efficiency of Posner’s networks calculated as follows: alerting, “No cue minus Neutral cue condition”; Orienting, “Neutral cue minus Spatial cue condition”; Executive, “Incongruent–Congruent Target condition" (see Methods)

^i^ IVRT—intraindividual variation of reaction time

In **bold**: statistically significant differences

The two groups, children with ADHD and controls, were statistically different regarding AC (0.93 ± 0.10 in controls higher than 0.90 ± 0.08 in ADHD, p = 0.04) and IVRT (0.16 ± 0.06 in controls lower than 0.23 ± 0.11 in ADHD, p = 0.04) of AllCD (Table 1), but not regarding RT. Among the other significant differences observed between groups (Table 1), highlights are the efficiency of the executive network regarding AC (-0.06 ± 0.06 in controls higher than -0.11 ± 0.08 in ADHD, p = 0.04), and the efficiency of the alerting network (0.18 ± 0.06 in controls lower than 0.30 ± 0.15 in ADHD, p = 0.007), and the efficiency of the executive network (0.19 ± 0.08 in controls lower than 0.29 ± 0.15 in ADHD, p = 0.018) regarding IVRT.

In the group with all 39 subjects, a strong correlation was observed between the total DSM score and the efficiencies of the alerting and executive networks (r = 0.62 and r = 0.54, respectively), and a moderate correlation was observed with the efficiency of the orienting network (r = 0.48) concerning IVRT (Table 2). Criteria for hyperactivity+impulsivity showed a strong correlation with the efficiency of the alerting network (r = 0.51) and a moderate correlation with the efficiency of the executive network (r = 0.40), respectively. The criteria for inattention showed moderate correlation with the efficiencies of alerting (r = 0.50), orienting (r = 0.41), and executive networks(r = 0.46). Overall, these correlations were stronger inside the ADHD group (Table 2). We did not observe significant correlations between behavioral dimensions (AC and RT) and ADHD DSM criteria.

**Table 2:** Correlation between DSM scores of ADHD and intraindividual variation in reaction time (IVRT) in ANT.

DSM criteria	IVRT		Control (n = 20)		ADHD (n = 19)		All subjects (n = 39)	
			ρ	p-value(ρ)	ρ	p-value(ρ)	r	p-value (r)
Inattention	All Conditions	0.21	0.368	0.33	0.164	0.40	0.011
	**Alerting**	0.16	0.493	0.31	0.195	**0.50**	**0.001**
	Orienting	0.27	0.255	0.34	0.153	0.41	0.010
	Executive	0.30	0.192	0.33	0.174	0.46	0.003
Hyperactivity + Impulsivity	All Conditions	0.08	0.745	0.48	0.038	0.38	0.017
	**Alerting**	0.22	0.349	0.33	0.170	**0.51**	**0.001**
	Orienting	0.22	0.356	0.34	0.156	0.34	0.034
	Executive	0.16	0.490	-0.14	0.562	0.40	0.011
Total	**All Conditions**	0.34	0.148	**0.51**	**0.027**	**0.50**	**0.001**
	**Alerting**	0.22	0.341	**0.59**	**0.008**	**0.62**	**< 0.001**
	Orienting	0.37	0.111	0.44	0.060	0.48	0.002
	**Executive**	0.40	0.079	0.44	0.056	**0.54**	**< 0.001**

ρ - Spearman's correlation coefficient; r—Pearson's correlation coefficient; IVRT—intraindividual variation of reaction time; **In bold: strong correlations (coeff. ≥ 0.50);** See Table 1

Thus, the ADHD subjects showed lower efficiencies of the executive and alerting networksregarding AC and IVRT, and this effect was correlated with DSM.

### ANT-related potentials

Wave morphology in the ANT-RPs for AllCd was nearly the same among groups although it significantly changed between channels (Fig 2) Due to the temporal dissociation between flank distractors and target stimuli, we observed potentials related to these distractors within the first 100 ms after target onset. In nearly all channels (Fig 2), we observed a group of ERPs related to Cue onset, a group of waves related to consecutive flank distractors and target onsets, and a slow variation in negative voltage between the cue and corresponding target, a Contingent Negative Variation (CNV).

The maximum amplitudes of late Target RPs, which correspond to P3, observed in the Pz parietal channel in all conditions as well as in AllCd, were higher in the control group than in the ADHD group (for AllCd, 14.26 ± 5.76 μV and 10.45 ± 6.26 μV, respectively; p = 0.010) (Fig 2 and Table 3). No significant correlations were found between the maximum amplitudes analyzed and DSM scores (inattention, hyperactivity+impulsivity, or total).

**Table 3:** Comparisons of maximum P3 peak amplitude (μV) in the Pz channel between groups in all ANT cue and target conditions and the mean value of all conditions.

ANT condition	Control			ADHD			p(levene)^b^	Stat^c^	p	p (corr)^d^
	mean	SD	p(ShW)^a^	mean	SD	p(ShW)				
All conditions	14.26	5.76	0.001	10.45	6.26	0.158	0.493	Z = -2.59	0.010	0.018
No cue	14.89	4.02	0.191	10.74	6.97	0.006	0.369	Z = -2.51	0.012	0.018
Neutral cue	14.66	6.48	0.009	12.53	6.09	0.012	0.793	Z = -2.12	0.034	0.034
Spatial cue	15.68	4.31	0.715	11.49	6.01	0.064	0.619	T = 2.51	0.017	0.020
Congruent target	15.05	6.27	0.002	12.12	5.18	0.033	0.757	Z = -2.71	0.010	0.018
Incongruent target	14.75	5.72	0.001	9.71	8.44	0.038	0.177	Z = -2.57	0.010	0.018

^a^ p-value related to Shapiro-Wilk Test for normality, applied in each group, by variable.

^b.^ p-value related to the Levene Test for homogeneity of variances, applied in groups, by variable

^c.^ Z—Mann-Whitney U test; T—Test for independent measures

^d.^ p-correction by Benjamini-Hochberg procedure (non independent measures).

See Table 1

### Interhemispheric asymmetries and their correlation with DSM

In ANT-RPs of AllCd, we found significant interhemispheric differences only in the temporal areas (F8xF7, T4xT3, and T6xT5) of both groups (Fig 3 and S1 Fig for a detailed view). In the control group, the point-to-point differences had higher level of significance (p<0.01) for the most part and they covered nearly the entire group-average signal duration. In the ADHD group, we observed differences only when comparing F8xF7 and T4xT3, mainly restricted to the late portions of CNV, first 240 ms after target onset, and after 770 ms.

Using FDR-corrected scanning, we compared the asymmetries related to different ANT conditions in each group resulting from subtracting the left hemisphere signals from the right hemisphere signals (Fig 4, amplitudes are normalized). There were no significant differences in interhemispheric asymmetry between any conditions in the ADHD group (Fig 4C and 4D). However, in the control group, there were significant differences in asymmetry magnitude between NoCue and NtCue conditions (p < 0.05), always with right-side prevalence. These differences encompassed nearly the entire duration of the signal resulting from the T4-T3 subtraction, and part of the late Target-RP resulting from the F8-F7 subtraction (Fig 4A, detail in Fig 5). Thus, regarding the alerting network, ANT-RPs in ADHD had lower right-side amplitude prevalence in the temporal regions that was observed in the controls.

On the other hand, the mean values of the group resulting from the subtraction of the left-side data from the right-side data, shown in Fig 4, indicate that an apparent asymmetry in the frontal (F4-F3), central (C4-C3), parietal (P4-P3), and occipital (O2-O1) regions shortly after target onset (see the values below) appeared in any ANT conditions in the ADHD group (Fig 4C and 4D). Asymmetries of these latencies were not evident in the control group in the different ANT conditions (Fig 4A and 4B). Although the values of these asymmetries were not significantly different between the groups according to FDR-corrected scanning, intervals of interest were established according to their time positions in each channel, with two intervals of interest in the F4-F3 channel and one interval of interest in the other ones (Fig 5, marked with red dashed rectangles).

**Fig 6:**
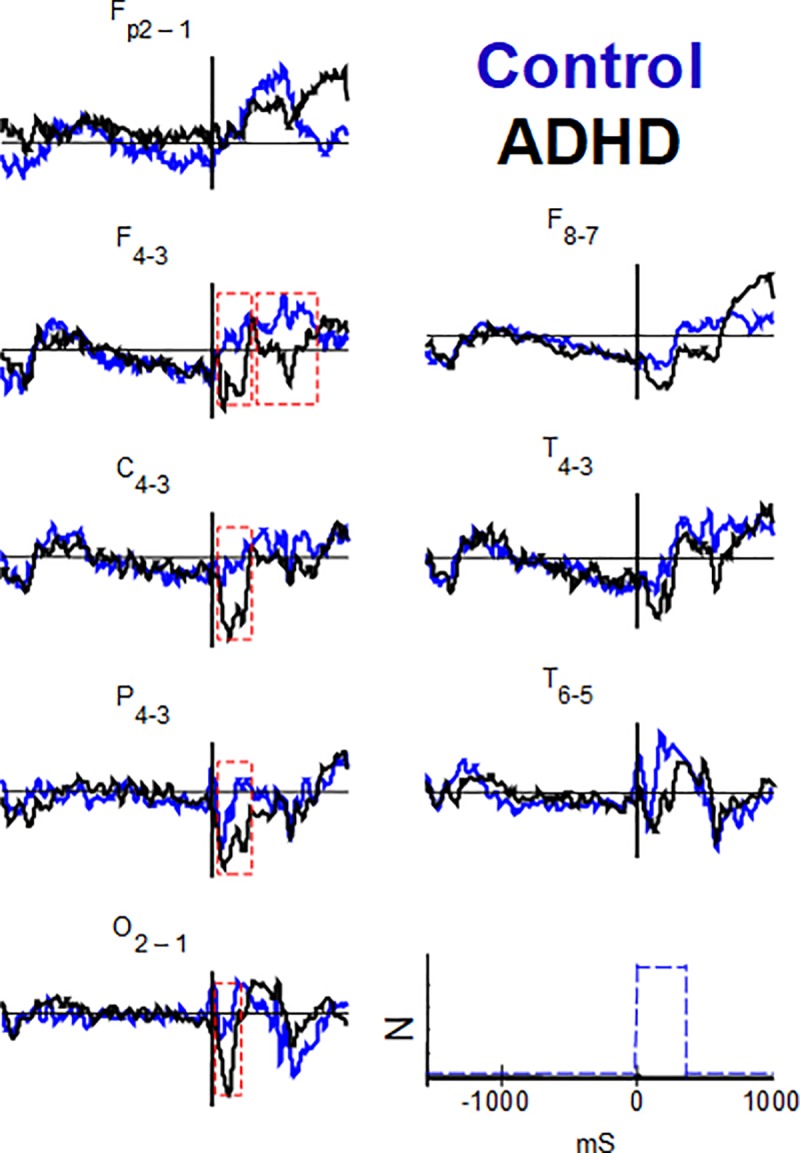
Interhemispheric differences (asymmetries) in the mean values of all conditions in ADHD and control groups. Control and ADHD groups in blue and red waves, respectively. The asymmetries of interest are delimited by dashed red boxes. Amplitudes are normalized (positive values above abscissae). See Fig 2, for channel labeling.

**Fig 7:**
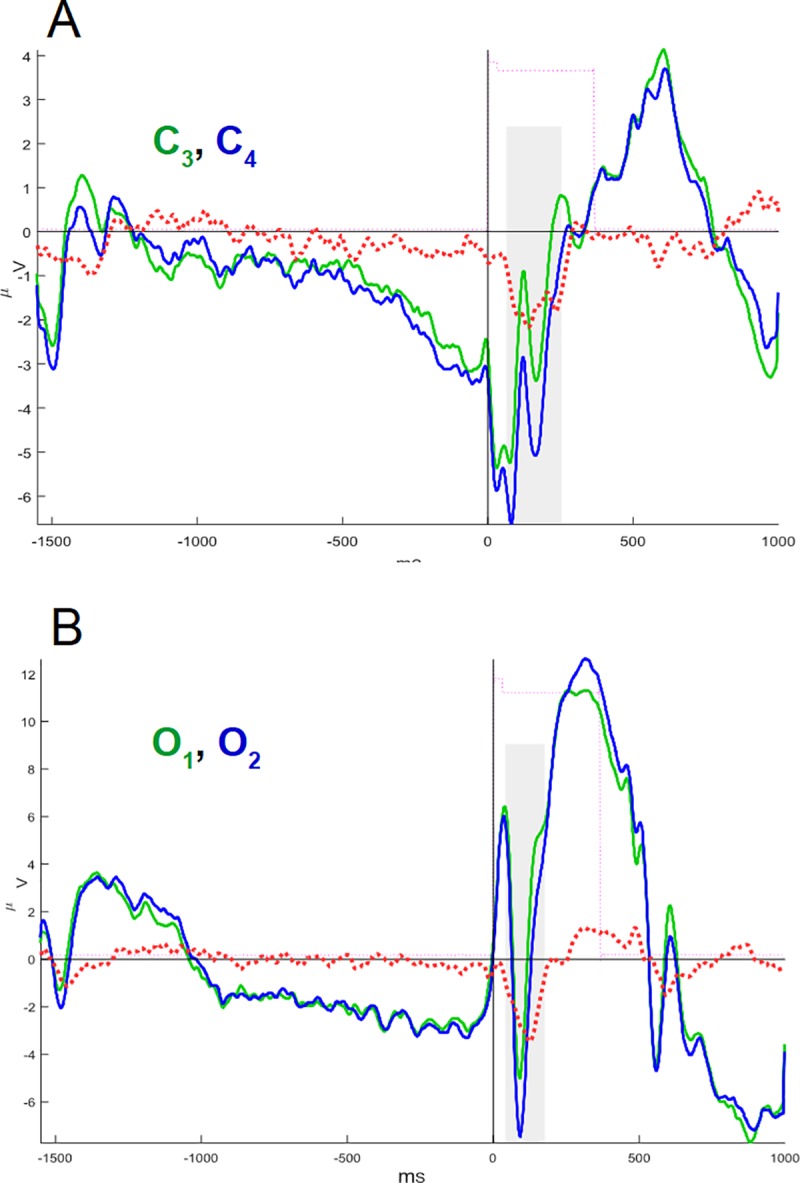
ANT-related potentials of central and occipital channels and their differences in the ADHD group. (A) ANT-related potentials in the central region. Superimposed potentials from the left (C3, green) and right (C4, blue) leads, and the arithmetic difference between them, revealing the rightward asymmetry (C4 –C3, dashed red line). (B) The same for potentials in occipital region from the left (O1) (green) and right (O2) (blue) leads. Amplitudes in μV.

Mean asymmetry amplitudes of the above intervals of interest only proved to be significantly different between groups in the central and occipital areas. The ADHD group had higher negativity amplitude than the control, regarding *AllCd* (-1.41 ± 1.88 μV and -0.22 ± 2.32 μV, respectively, p = 0.049) and incongruent target distractors (-1.50 ± 2.00 μV and -0.10 ± 2.29 μV, respectively; p = 0.048) in C4-C3, in the window from 45 to 290 ms, showing a higher right-side amplitude prevalence of negative signal in the central region in ADHD compared to the controls (Fig 6 and Fig 7A). We also observed a significant difference between groups in O2-O1, in the window between 40 and 200 ms, emphasizing that the ADHD group expresses higher mean negativity to the right than the control group (Fig 6 and Fig 7B): AllCd (-1.88 ± 2.09 μV and -0.22 ± 2.07 μV, respectively; p = 0.026), NoCue (-1.98 ± 2.32 μV and -0.24 ± 1.79 μV, p = 0.029), NtCue (-1.92 ± 2.36 μV and -0.40 ± 2.00 μV, p = 0.035), SpCue (-1.74 ± 1.90 μV and -0.04 ± 2.79 μV, p = 0.034), CgnTg (-1.76 ± 2.04 μV and -0.19 ± 2.16 μV, p = 0.032) and incongruent target distractors (-2.00 ± 2.24 μV and -0.26 ± 2.08 μV, p = 0.036) (Table 4). Thus, in any ANT condition, ADHD subjects showed significantly higher asymmetries in the occipital regions 40–200 ms after target onset than controls. In the central regions, higher asymmetries in ADHD were observed for AllCd and incongruent target condition.

**Table 4:** Comparisons of interhemispheric asymmetry (mean amplitude) between groups.

ANT condition	Asymmetry in intervals of interest	Control			ADHD			p (levene)	Stat	p-value	p (corrected)
		mean	SD	p (ShW)	mean	SD	p (ShW)				
**All conditions**	**C4-C3** **(45–290 ms)**	**-0.20**	**2.32**	**0.011**	**-1.41**	**1.88**	**0.000**	**0.008**	**Z = 1.96**	**0.049**	0.147
No cue		-0.33	2.59	0.025	-1.46	2.14	0,000	0,052	Z = 1.33	0.182	0.218
Neutral cue		-0.17	2.52	0,008	-1.25	1.85	0,001	0,013	Z = 1.13	0.255	0.255
Spatial cue		-0.12	2.45	0,027	-1.54	1.86	0,000	0,014	Z = 1.78	0.074	0.148
Congruent target		-0.31	2.52	0,042	-1.33	1.86	0,000	0,003	Z = 1.47	0.140	0.210
**Incongru-ent target**		**-0.10**	**2.29**	**0,001**	**-1.50**	**2.00**	**0,000**	**0,013**	**Z = 1.98**	**0.047**	0.147
**All Conditions**	**O2-O1** **(40–200 ms)**	**-0.22**	**2.07**	**0.002**	**-1.88**	**2.09**	**0.030**	0.254	**Z = 2.21**	**0.026**	**0.036**
**No cue**		**-0.24**	**1.79**	**0,001**	**-1.98**	**2.32**	**0,049**	0,845	**Z = 2.17**	**0.029**	**0.036**
**Neutral cue**		**-0.40**	**2.00**	**0,024**	**-1.92**	**2.36**	0,064	0,247	**T = 2.18**	**0.035**	**0.036**
**Spatial cue**		**-0.04**	**2.79**	**0,005**	**-1.74**	**1.90**	**0,017**	**0,001**	**Z = 2.12**	**0.034**	**0.036**
**Congru-ent target**		**-0.19**	**2.16**	**0,001**	**-1.76**	**2.04**	**0,033**	**0,046**	**Z = 2.14**	**0.032**	**0.036**
**Incongru-ent target**		**-0.26**	**2.08**	**0,002**	**-2.00**	**2.24**	**0,018**	**0,530**	**Z = 2.09**	**0.036**	**0.036**

In bold: Statistically significant differences; See Tables 1 and 3

**Fig 8:**
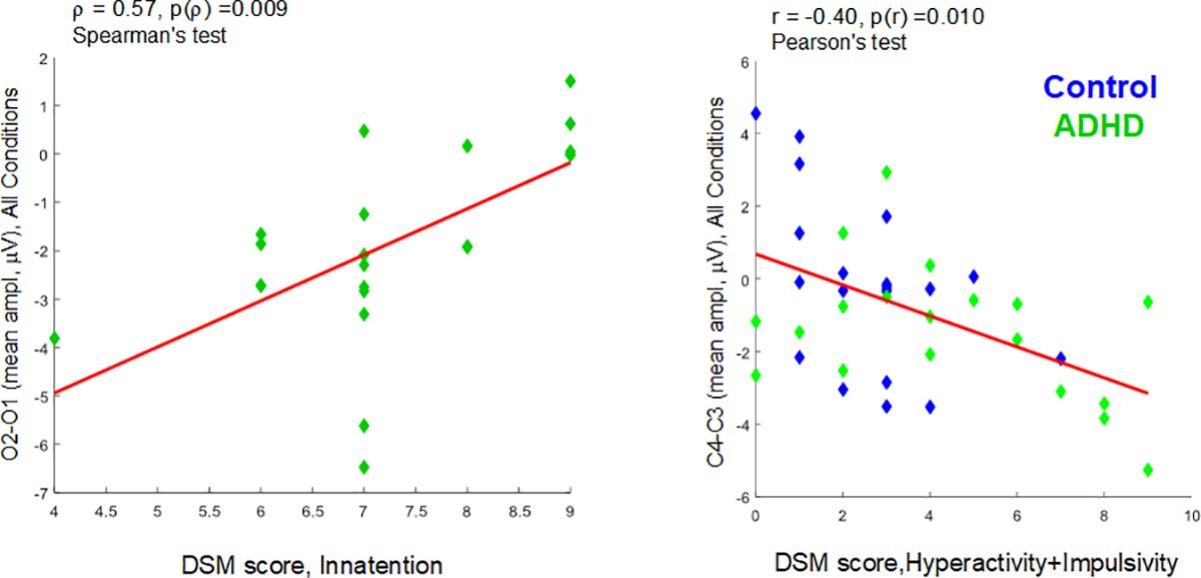
Scatter plots and linear regression models of correlation between asymmetries and DSM scores. Left: mean amplitudes of O2-O1 asymmetry of all conditions (AllCd) (40–200ms after target onset) versus DSM-Inattention scores in the ADHD group. Right: mean amplitudes of C4-C3 asymmetry of AllCd (45–290ms after target onset) versus DSM hyperactivity+Impulsivity scores in all subjects (blue, Control group; green, ADHD group).

These asymmetries were correlated with DSM scores in ADHD. In the ADHD group, mean amplitudes in O2-O1 asymmetry related to any ANT conditions and AllCd showed a strong correlation with criteria for inattention (from ρ = 0.52 to ρ = 0.75, Table 5), so that the higher the negativity of the right occipital area compared to the left, the better the attention in ADHD (Table 5, Fig 8). In all the 39 subjects gathered, mean amplitudes in C4-C3 related to any ANT conditions and AllCd showed correlation with criteria for hyperactivity+impulsivity (from r = -0.40 to r = -0.50), so much so that regardless of an ADHD diagnosis, the more hyperactive and impulsive the behavior, the higher the amplitude prevalence of the right-side negative wave over the left in the central region.

**Table 5:** Correlations between asymmetries (mean amplitude) in occipital and central regions of different ANT conditions and DSM scores.

DSM criteria	Asymmetry in intervals of interest	ANT conditions	Control		ADHD		All subjects	
			ρ	p-value(ρ)	ρ	p-value(ρ)	r	p-value (r)
Inattention	O2-O1 (40–200 ms)	All Conditions	0.10	0.666	**0.58**	**0.009**	-0.16	0.317
		No cue	0.07	0.758	**0.52**	**0.023**	-0.19	0.241
		Neutral cue	0.18	0.447	**0.54**	**0.018**	-0.12	0.459
		Spatial cue	0.10	0.690	**0.75**	**0.000**	-0.15	0.354
		Congruent target	0.12	0.613	**0.63**	**0.004**	-0.14	0.385
		Incongruent target	0.11	0.645	**0.55**	**0.015**	-0.18	0.280
	C4-C3 (45–290 ms)	All Conditions	-0.26	0.267	0.03	0.915	-0.30	0.064
		No cue	-0.15	0.538	0.12	0.618	-0.21	0.192
		Neutral cue	-0.28	0.225	0.03	0.915	-0.28	0.082
		Spatial cue	-0.27	0.247	0.07	0.779	-0.35	0.028
		Congruent target	-0.18	0.439	0.11	0.639	-0.22	0.184
		Incongruent target	-0.38	0.102	0.04	0.885	-0.36	0.022
Hyperactive + Impulsive	O2-O1 (40–200 ms)	All Conditions	-0.26	0.260	0.19	0.424	-0.14	0.395
		No cue	-0.22	0.359	0.18	0.463	-0.14	0.389
		Neutral cue	-0.21	0.366	0.24	0.317	-0.04	0.812
		Spatial cue	-0.22	0.344	-0.02	0.920	-0.21	0.201
		Congruent target	-0.28	0.229	0.22	0.358	-0.07	0.662
		Incongruent target	-0.21	0.377	0.07	0.780	-0.20	0.224
	C4-C3 (45–290 ms)	All Conditions	**-0.51**	**0.021**	-0.31	0.200	-0.49	0.002
		No cue	**-0.55**	**0.012**	-0.43	0.063	**-0.50**	**0.001**
		Neutral cue	-0.39	0.089	-0.20	0.403	-0.40	0.011
		Spatial cue	-0.41	0.074	-0.34	0.150	-0.46	0.003
		Congruent target	**-0.51**	**0.021**	-0.27	0.256	-0.46	0.003
		Incongruent target	-0.42	0.068	-0.29	0.221	-0.48	0.002
Total	O2-O1 (40–200 ms)	All Conditions	-0.04	0.873	0.44	0.057	-0.17	0.299
		No cue	-0.03	0.886	0.42	0.077	-0.19	0.243
		Neutral cue	0.03	0.886	0.48	0.036	-0.09	0.601
		Spatial cue	0.00	0.997	0.25	0.305	-0.20	0.216
		Congruent target	-0.03	0.899	0.47	0.040	-0.12	0.468
		Incongruent target	0.01	0.972	0.34	0.153	-0.21	0.195
	C4-C3 (45–290 ms)	All Conditions	**-0.48**	**0.031**	-0.23	0.338	-0.46	0.003
		No cue	-0.42	0.066	-0.32	0.175	-0.41	0.009
		Neutral cue	**-0.45**	**0.048**	-0.13	0.592	-0.41	0.010
		Spatial cue	-0.44	0.052	-0.24	0.314	-0.48	0.002
		Congruent target	-0.44	0.051	-0.18	0.467	-0.39	0.010
		Incongruent target	**-0.51**	**0.021**	-0.20	0.417	**-0.50**	**0.001**

In bold: strong correlations (coefficient ≥ 0.50); ρ - Spearman's correlation coefficient; r—Pearson's correlation coefficient; Non-corrected p-values

**Fig 9:**
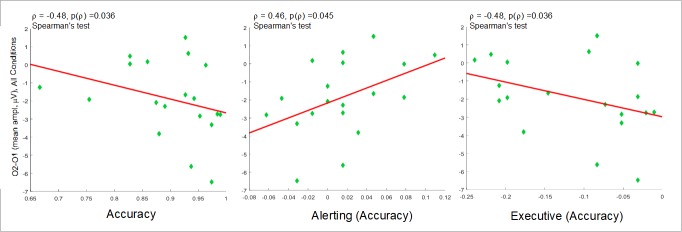
Correlation between O2-O1 asymmetry and ANT performance. O1-O2 (mean amplitude) of AllCd and accuracy (left), and efficiencies of the alerting (center) and executive networks (right) regarding accuracy.

As to attentional networks, only in the ADHD group the O2-O1 asymmetry showed moderate negative correlation with the AC total score (ρ = -0.48, p = 0.037) and the efficiency of the executive network over AC (ρ = -0.48, p = 0.037), and moderate positive correlation with the efficiency of the alerting network over AC (ρ = 0.46, p = 0.045) (S2 Table and Fig 9). This means that only in ADHD, the occipital asymmetry affects ANT performance, so that the higher the right-side negativity compared to the left, the better AC, the higher the efficiency of the executive network related to AC and the lower the efficiency of the alerting network related to AC. See all correlations in S2 Table.

In general, the rightward occipital asymmetry in ADHD subjects proved to be inversely proportional to DSM scores of inattention and directly proportional to executive accuracy and efficiency.

The number of left- and right-handed subjects did not correlate with any of the asymmetries studied.

## Discussion

Behavioral and neurophysiological ANT findings related to ADHD in the present study are in accordance with previous publications [15–17,31, 32]. When we evaluated the interhemispheric asymmetries of ANT-RPs and their correlation with attentional networks, we found smaller asymmetry in ADHD than in typically developing subjects related to efficiency of the alerting network. On the other hand, an asymmetry caused by a higher amplitude of early negative components in the right occipital region, which were inversely correlated to efficiency of the alerting network and proportional to AC and to efficiency of the executive network regarding accuracy, emphasized that there might be potential functional compensatory mechanisms, which were restricted to inattention symptomatology, as scrutinized by DSM [2,3].

Unlike the above-mentioned studies, we homogenized our samples by gender and age, and consequently, we worked with relatively homogeneous EEG patterns, as it is only from the age of 11 that the EEG may be considered homogeneous in terms of development [41]. We therefore included only boys of a narrow age range and considered confounders that are usually ignored, such as playing video and PC games, which might develop skills to which ANT is sensitive by nature, and the interference of sleep deprivation [42]. The exclusion of subjects due to comorbidities was a process of great sensitivity, as we defined a rule that participants suspected of having comorbid conditions that are believed to be relevant should be removed, since they are very prevalent in ADHD and consist of important confounders [43, 44] (see exclusion criterion ‘3’ in Methods). Unfortunately, the rigor of the exclusion criteria resulted in a substantial limitation in this study, which was the small size of the final sample used in the analysis procedures. Aside from statistical issues regarding the comparison between the two independent groups, this rendered impossible any inference about functional and behavioral correlates in smaller samples of ADHD subtypes. On the other hand, due to this rigorous sampling strategy, the groups are quite consistent concerning the primary object of the study, which is ADHD. The lower IQ found in the ADHD group can be considered a bias. However, it could also be an artifact of the clinical condition studied. It has been described in literature that WISC performance is affected by attention deficit [45, 46], which could have contributed to the results in our ADHD group. Furthermore, all the boys had IQ scores within normal range. For I.Q. estimation, we selected subtests of WISC-III, which are reference in the Brazilian population [35], as we value cultural influences [47].

### ADHD diagnosis and ANT performance

In the present study, we found strong and moderate correlations between behavioral/biological dimensions and DSM. In a concomitant study, we reclassified these subjects based on a multivariate analysis of a dataset resulting from ANT application. We observed a “substantial level of agreement” between DSM and the phenomenological dimensions of ANT (behavioral, psychological, and neurophysiological, similar to what is described in the present study): kappa index = 0.72 [48], and the specificity and sensitivity of the DSM-IV-TR regarding biological variables were 75% and 89%, respectively [49].

According to the results obtained, the ADHD group showed lower efficiencies of the alerting and executive networks regarding IVRT and AC. Previous studies corroborate these findings, as patients with ADHD show lower AC and higher IVRT [14, 17, 31, 32] than controls, due to a lower efficiency of the executive network.

According to previous findings [14–17], orienting network does not seem to influence ADHD behavior. In the present study, DSM scores showed correlation mostly to the efficiency of the alerting network, especially in the ADHD group, followed by the efficiency of the executive network, thus suggesting an essential role of alertness in ADHD clinical condition. Our findings reinforce the importance of alerting network in ADHD.

### Correlations of ANT-RPs with ANT performance and DSM

In the ANT paradigm that was used here [31, 32, 37], there is a temporal dissociation of flank distractors and target onset at 100 ms. The temporal dissociation of the target seems to reduce the damaging effect of flank distractors on behavior, which is higher in the case of incongruence between target and distractors [50]. Therefore, the present version of the ANT would be more specific and sensitive to observe inherent differences between ADHD and control subjects. However, temporal dissociation prevents us from relating the characteristics of average-latency potential to a specific stimulus due to the overlapping of ERP responses to both dissociated stimuli. We cannot estimate how much the potentials observed correspond to the classic components P1 and N2 [51], despite the fact that latencies are compatible with the appearance of flanks (100 and 200 ms). The second negativity could result from the combination of flank N2 with target P1. Therefore, our direct measures were limited to the P3 component, as in Kratz et al. [31].

Observing the P3 wave in ADHD, we obtained results that generally replicate literature [52], especially the findings by Kratz et al using ANT [31]: the ADHD group showed reduced P3 wave amplitude in all ANT conditions, with no specific relationship with attentional networks. Overall, P3 wave reflects the temporo-parietal high-order information processing, which is under the control of frontal executive centers [53, 54]. As cue and target conditions in the present study were equiprobable, we can consider that P3 possibly reflected the allocation of information workload for target visuospatial processing [37, 53]. Therefore, the lower P3 amplitude in ADHD was possibly a result of impaired cognitive processes related to visuospatial information processing in ANT.

When analyzing asymmetries in ANT-RPs of AllCd, significant interhemispheric differences were observed in the ADHD group only in the mid- and anterior temporal areas, where they showed a right-side prevalence. In the control group, this prevalence in the same areas presented a higher level of significance and time duration and it was also observed in the posterior temporal region. When we considered asymmetries for different cue and target conditions in terms of attentional networks calculated according to Fan et al. [12], results only showed a significant effect on the characteristics of the efficiency of the alerting network and exclusively in the control group, with predominance of amplitudes of the right mid- and anterior temporal potentials. We found no significant asymmetries regarding orienting and efficiency of the executive network in the two groups, either. The different behavior of neurophysiological correlates of the efficiency of the alerting network in the groups reinforced the idea that changes in this network, which controls and modulates sustained attention [20], would play an essential role in ADHD physiology. Moreover, our results seem to corroborate the topography of this effect, pinpointing it in the right temporal lobe [55].

Evidence found after the publication by Posner and Petersen [10] has reviewed the original model of alerting network, suggesting there is segregation of its phasic and intrinsic (tonic) components in the left and right hemispheres, respectively [11, 20, 25]. It has been suggested that the activity on the left, associated to phasic alertness, would correspond to selective voluntary attention mechanisms triggered by attention modulation sustained by the warning signal [56]. However, recent studies on brain changes caused by lesions [28], with functional neuroimage [27], and ERPs with repetitive transcranial magnetic stimulation [26] have also suggested the participation of the right hemisphere in phasic alertness, clearly showing that the model for this attentional network is still not definitive. Our findings corroborate the participation of the right hemisphere in phasic alertness, and suggest that its mechanisms would be corrupted in ADHD, which could be a substrate for behavioral differences found between the groups regarding of the efficiency of the alerting network.

In ADHD, irrelevant information seem to overwhelm brain activity, leading to impaired regulation of voluntary attentional spotlight according to task demand [57], suggesting a dysfunction in selective and sustained attention, which is related to lower efficiency of the alerting network. In a series of studies across verbal paradigms such as continuous performance test, Hale et al. [58–61] systematically show functional asymmetries, with higher activity of the right hemisphere in ADHD during tasks that evoke modulation of voluntary sustained attention, which is normally considered to be related to the left hemisphere and based on verbal functions [62, 63]. These findings by Hale et al. are probably related to some compensatory processes in ADHD. We can speculate that some right-hemisphere mechanisms in the points homologous to the left ‘verbal’ zones in ADHD, which are normally activated during verbal tasks, could be used as a compensatory system to mitigate verbal cognition problems, which are known to be typical of ADHD [9]. Similarly, patients with motor aphasia due to lesions in the left frontal area have been observed to improve speech communication through musical prosody, which is considered as a function of the right frontal cortex that corresponds to verbal-related functions of the homologous left region [64, 65]. In ANT responses, these right-hemisphere mechanisms were apparently not involved since ANT is a set of non-verbal tasks, explaining the rightward asymmetry found in the controls. In another study by our laboratory (with the same subjects), analyzing the ongoing EEG, ADHD subjects showed certain signs of reduced activation of the left temporal and frontal areas during relaxed wakefulness and intermittent photic stimulation, without verbal tasks or any other tasks demanding voluntary attention [55].

Possible compensatory mechanisms in ADHD were evident in this study due to a higher negativity in the right occipital region than in the left, evoked in the first 200 ms of target processing. It is well known that the visual processing that subsides perception is modulated by top-down attention [66]. Early ERPs, such as P1 and N2, are related to activity in visual areas and modulated by cue information [67, 68], revealing top-down attention effect on visual perception [69]. In the ANT, the modulation of N2 amplitude has been previously related to Posner's alerting network, but not to the executive network [67]. On the other hand, bottom-up attention also drives visual perception, however inespecifically, triggered by pop-out stimuli [69]. The above-mentioned asymmetry appeared in all cue and target conditions, suggesting its non-specificity regarding Posner's attentional networks and their specific mechanisms for attentional modulation of neural processes. Thus, there would be a bottom-up attention process of visual sensorial adaptation, which is specific to ADHD and that causes this asymmetry, either locally or driven by other associative systems.

We emphasized the compensatory nature of this asymmetry with right-side prevalence when we observed that its magnitude is inversely proportional to the inattention score and directly proportional to the accuracy of ANT responses. Asymmetry magnitude is also directly proportional to the efficiency of the executive network, and inversely proportional to the efficiency of the alerting network, both related to accuracy. This finding suggests that despite the top-down attention impairment related to alerting network, a bottom-up compensatory mechanism, probably located in early visual system, supplied visual information to improve conflict resolution between target and distractors and to provide an adequate behavioral response.

Studies have suggested that posterior regions seem to develop compensatory functional mechanisms for the low performance of prefrontal executive processes, which participate in sustained attention control [8, 9]. Somehow, due to lower alertness efficiency, the ADHD brain seemed to recruit local resources for perception and representation in the visual cortex independent of attentional networks, thus improving accuracy in the responses and final performance in conflict resolution.

As studies have reported, attention is modulated by the interplay between dopaminergic and noradrenergic systems in the prefrontal cortex, and it seems that these neurotransmitters perform different roles in attention-dependent behavioral performance in ADHD: the number of hits and the IVRT are maximized by atomoxetine and methylphenidate, respectively [32, 70]. Thus, a correlation between accuracy and alerting network can be established.

In this context, we have proposed that impairment of the alerting network could primarily affect target perception causing lower accuracy (lower hit rates). On the other hand, bottom-up asymmetrical compensatory processes at an early stage of visual perception, which are known to be reflected in visual P1-N2 potentials, could improve ANT hit rates, and consequently, the performance of the executive network, especially concerning accuracy.

Unlike occipital asymmetry, asymmetry with right-side prevalence detected in the central region (C4-C3) within the 45–290 ms window after target onset showed to be significantly higher in ADHD only compared to AllCd and regarding the ‘incongruent target distractor’ condition. We demonstrated that IVRT is higher in ADHD in conflict conditions, revealed by the lower efficiency of the executive network related to motor control. Studies show that ADHD subjects usually have higher IVRT [71]. Our behavioral findings suggest that motor control is poorer in ADHD and central asymmetry seems to be a neurophysiological correlate of the selective inhibition impairment observed by Yordanova et al. [72]. Therefore, asymmetry is positively correlated with DSM scores, especially those of hyperactivity+impulsivity. However, this correlation is only significant considering all subjects (and in some cases, only subjects from the control group), showing that it is group-unspecific. A study showed significant correlation between impulsivity and ADHD scores in typical adults with higher-volume anatomic asymmetry in the right caudate nucleus compared to the left, thus emphasizing the dimensional character of phenomena related to control mechanisms of the motor behavior [28]. Similar anatomic asymmetry has also been observed in children with ADHD [4–6]. In ADHD, an alteration in motor inhibitory control has been shown during the presentation of irrelevant stimuli in attentional tasks [72] correlated to clinical and performance scores in terms of AC and IVRT [73]. IVRT was also inversely correlated to a lower prefrontal activity in ADHD [74]. Central asymmetry with right-side prevalence, which might also mean some insufficiency of the left-side functional status, might correspond to inefficient voluntary attention and behavior modulation in fronto-striatal circuits, a phenomenon that is not specific to ADHD neurophysiology, but that seems to have been potentiated in this disorder.

### Conclusion: ADHD and alertness

Based on the findings discussed here, we defend that the efficiency of the alerting network affects clinical characteristics related to inattention in ADHD, and this inefficiency could compromise visual cognition, resulting in a poorer executive performance, which was observed regarding both AC and IVRT. The likely compensatory mechanisms evidenced in the occipital region of individuals with ADHD would be quite effective to optimize AC and performance in conflict resolution with lower efficiency of the alerting network, suggesting an impaired primordial alerting function, which induces a collateral adaptive process. While IRVT depends on the modulatory control of motricity [73], the number of hits in attentional tasks obviously depends on the perception of target characteristics and on the surroundings, feeding the executive network with information for conflict detection and resolution. Thus, it is possible that the primary alteration in ADHD is triggered by alerting network drivers, which modulates perception, while the executive impairment related to decision making based on perceptual information may arise from this alteration.

Occipital and central asymmetries would be relatively independent phenomena from the clinical condition and from primordial ADHD mechanisms. Central asymmetry showed to be dimensional (of a different nature from occipital asymmetry), perhaps related to the performance of executive control over behavior, while coexisting occipital asymmetry showed to be a more specific condition related to ADHD phenomenology. This complex model supports the two-dimensional ADHD clinical phenomenology.

The variability of ADHD clinical phenomenology, defined by at least three consistent subtypes (inattentive, hyperactive/impulsive, and combined), leads us to speculate about the coexistence of different neural processes, which would be specific to each subtype, with mechanisms common to all of them. Perhaps the ADHD subtypes correlate with alerting and executive networks in different ways. We shall establish these correlations in further studies with larger samples of ADHD subjects.

## Supporting information

S1 Dataset ANT-related potentials Data structure holding all waves of the 39 subjects (control = 20, ADHD = 19), for the 20 channels and five ANT conditions plus grand average of all conditions (a total of 9360 waves with 1531 bins, at a sampling rate of 600 bins/second). See dedails in S2 Dataset.
(MAT)

S2 Dataset Behavioral and ERP data. Tables with ANT performances, DSM and WISC scores, confounding variables, and peak and mean amplitudes for P3 and asymmetries respectively. Description of the data structure in S1 Dataset.
(XLS)

S1 Fig Comparisons between temporal regions in control and ADHD groups. Detail from Fig 3, highlighting significant statistical differences between hemispheres (see the caption of Fig 3).
(TIF)

S1 Table Correlations between DSM scores of ADHD and confounder variables and demographic variables. Total, inattention—Inn, and hyperactivity+impulsivity–Imp+hyp scores. Pearson’s correlation test (r) for all subjects.
(XLS)

S2 Table Correlations between performance in ANT and occipital asymmetry. O2 –O1 (40–200 ms, mean amplitude) for each ANT condition correlated to Accuracy scores, and also efficiencies of the alerting and executive networks (ANT conditions: NtCue, neutral cue; SpCue, spatial cue; NoCue, no cue; CngTg, congruent target; IncTg, incongruent target; and AllCd, all conditions). For each group: Spearman’s test; and for all subjects: Pearson’s test.
(XLS)
