# Supplementary figures and images for: Neurophysiological and behavioral correlates of alertness impairment and compensatory processes in ADHD evidenced by the Attention Network Test
Source: PLoS One. 2019 Jul 25;14(7):e0219472. doi: 10.1371/journal.pone.0219472 (PMC6657843; doi:10.1371/journal.pone.0219472)

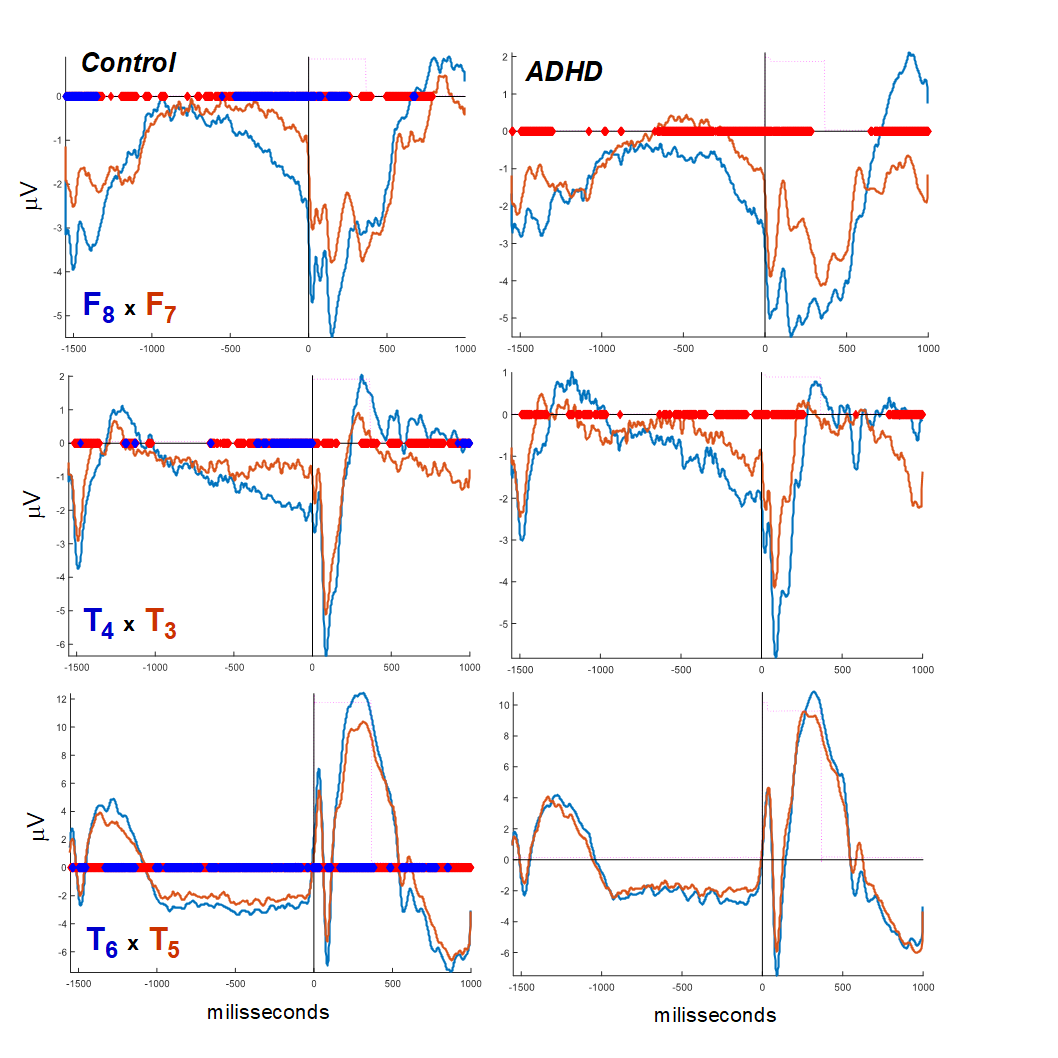

Supplement: S1 Fig — Detail from Fig 3, highlighting significant statistical differences between hemispheres (see the caption of Fig 3). (TIF) [file pone.0219472.s003.tif]
